# Internet-based treatment of stress urinary incontinence: a randomised controlled study with focus on pelvic floor muscle training

**DOI:** 10.1111/j.1464-410X.2012.11713.x

**Published:** 2013-01-25

**Authors:** Malin Sjöström, Göran Umefjord, Hans Stenlund, Per Carlbring, Gerhard Andersson, Eva Samuelsson

**Affiliations:** Department of Public Health and Clinical Medicine, Umeå UniversityUmeå, Sweden; * Department of Psychology, Stockholm UniversityStockholm, Sweden; † Department of Behavioural Sciences and Learning, Linköping UniversityLinköping, Sweden

**Keywords:** stress urinary incontinence, randomised controlled study, Internet, pelvic floor muscle training, self-management, cognitive behavioural therapy

## Abstract

**What’s known on the subject? and What does the study add?:**

- Stress urinary incontinence (SUI) affects 10–35% of women, and it is sometimes very distressful. Pelvic floor exercises are the first line of treatment, but access barriers or embarrassment may prevent women from seeking help. There is a need for new, simple, and effective ways to deliver treatment.
- Management of SUI without face-to-face contact is possible, and Internet-based treatment is a new, promising treatment alternative.

**Objective:**

- To compare two treatment programmes for stress urinary incontinence (SUI) without face-to-face contact: one Internet-based and one sent by post.

**Patients and Methods:**

- Randomised, controlled trial conducted in Sweden 2009–2011. Computer-generated block-randomisation, allocation by independent administrator. No ‘blinding’.
- The study included 250 community-dwelling women aged 18–70 years, with SUI ≥1 time/week. Consecutive online recruitment.
- The women had 3 months of either; (i) An Internet-based treatment programme (124 women), including e-mail support and cognitive behavioural therapy assignments or (ii) A treatment programme sent by post (126). Both programmes focused mainly on pelvic floor muscle training.
- Primary outcomes: symptom-score (International Consultation on Incontinence Questionnaire Short Form, ICIQ-UI SF) and condition-specific quality of life (ICIQ-Lower Urinary Tract Symptoms Quality of Life, ICIQ-LUTSQoL). Secondary outcomes: (i) Patient Global Impression of Improvement, (ii) Incontinence aids, (iii) Patient satisfaction, (iv) Health-specific QoL (EQ5D-Visual Analogue Scale), and (v) Incontinence episode frequency. Follow-up after 4 months via self-assessed postal questionnaires.

**Results:**

- In all, 12% (30 women) were lost to follow-up. Intention-to-treat analysis showed highly significant improvements (*P* < 0.001) with large effect sizes (>0.8) with both interventions, but there were no significant differences between groups in primary outcomes. The mean (sd) changes in symptom-score were: Internet 3.4 (3.4), Postal 2.9 (3.1) (*P* = 0.27). The mean (sd) changes in condition-specific QoL were: Internet 4.8 (6.1), Postal 4.6 (6.7) (*P* = 0.52).
- Compared with the postal-group, more participants in the Internet-group perceived they were much or very much improved (40.9% (43/105) vs 26.5% (30/113), *P* = 0.01), reported reduced usage of incontinence aids (59.5% (47/79) vs 41.4% (34/82), *P* = 0.02) and were satisfied with the treatment programme (84.8% (89/105) vs 62.9% (71/113), *P* < 0.001).
- Health-specific QoL improved in the Internet-group (mean change 3.7 (10.9), *P* = 0.001), but not in the postal-group (1.9 (13.0), *P* = 0.13).
- Overall, 69.8% (120/172) of participants reported complete lack of leakage or reduced number of leakage episodes by >50%.

**Conclusions:**

- Concerning primary outcomes, treatment effects were similar between groups whereas for secondary outcomes the Internet-based treatment was more effective.
- Internet-based treatment for SUI is a new, promising treatment alternative.

## Introduction

Stress urinary incontinence (SUI) is the involuntary leakage of urine when sneezing, coughing, or on exertion 1. Prevalence of SUI is 10–35% among women 2,3, and quality of life (QoL) may be impaired 4. Primary care professionals are usually the first to diagnose and treat the condition. Diagnosis can be based on structured history taking and bladder diaries 5. The recommended first-line treatment is pelvic floor muscle training 3,5–8, which leads to improvement or cure in two-thirds of patients and has no serious adverse effects 5,7,8. In addition, lifestyle changes (weight loss if body mass index >30 kg/m^2^, smoking cessation, reduction of fluid intake if high) may help 5–6, and a few small studies suggest that cognitive behavioural therapy may be useful in patients with incontinence 9,10. Despite the existence of effective treatments, only ≈20% of affected women seek medical care 11. There are several explanations for this: the leakage may not be a problem to the individual, it may be considered a part of normal ageing, expectations of successful treatments are low, patients may think they can manage on their own, or they may be too embarrassed to seek help 3. Also, access to care may be limited, depending on patients’ location and health care organisation, and SUI is often given a low priority in times of financial constraint. Moreover, once the woman seeks care, management is variable, and some women perceive that they do not get any help when consulting their physician 12. Such under-treatment may be due to a lack of confidence among healthcare providers in the management of UI 13, but could also be due to a lack of resources, as supervised pelvic floor muscle training is demanding of staff.

There is no consensus on how pelvic floor muscle training should best be performed 14. As a guideline, the National Institute for Health and Clinical Excellence recommends at least eight contractions three times daily during a 3-month period 7. Before training initiation, the strength of the pelvic floor muscle contraction should be digitally assessed 6, but it is unclear whether this enhances the effect 7. Supervised training sessions might give the largest improvements 14, but self-help booklets with instructions for training at home are often used in everyday practice, and have been shown to reduce the number of leakage episodes by 50% 15.

E-health is a growing field that offers new, flexible, and easily accessible treatment possibilities 16. Internet-delivered treatments have previously been developed and tested for several medical conditions, e.g. chronic pain, headache, irritable bowel syndrome, and obesity 17. Women are known to often use the Internet for health issues 18, to seek second opinions, due to discontent with healthcare providers, and for embarrassing conditions 19. Different methods for the delivery of SUI treatments, e.g. Internet-based or self-management, have been identified as an important research field 5. If they are found effective, such treatments could potentially increase access to care for many women. The aim of the present study was to compare the effect of two different treatment programmes for SUI without face-to-face contact: an Internet-based programme and a programme sent by post.

## Patients and Methods

We performed a randomised, controlled study with two open parallel treatment arms. In all, 250 community-dwelling women, aged 18–70 years, with SUI at least once weekly were recruited via our open access website, http://www.econtinence.se. Invitations to the study were published on national websites for medical advice, and as advertisements in daily newspapers. Table 1 reports inclusion and exclusion criteria.

**Table 1:** Inclusion and exclusion criteria.

Inclusion criteria	Exclusion criteria
Female	Pregnancy
Age 18–70 years	Previous UI surgery
SUI ≥1 time/week	Known malignancy in lower abdomen
Ability to read and write Swedish	Difficulties with passing urine
Access to computer with Internet connection	Macroscopic haematuria
	Intermenstrual bleedings
	Severe psychiatric disorders, or HADS score >15 for depression or anxiety
	Neurological disease with affection on sensibility in legs or lower abdomen

HADS, Hospital Anxiety and Depression Scale.

Women answered an online, 17-item survey with automated immediate response for initial screening of eligibility criteria. Items included questions on type of UI and the Incontinence Severity Index 20. Those found eligible were asked to register contact details and were sent a postal questionnaire for further evaluation. This included a detailed medical history, socio-economic data, lifestyle, Internet usage, motivation, symptoms of anxiety or depression (the Hospital Anxiety and Depression Scale [HADS]) 21, validated instruments for baseline investigation of outcome measures (see below), and a 2-day bladder diary (time and measured volume of micturition, time and estimated volume of leakage episodes). We (M.S. or E.S.) assessed all questionnaires, instruments, and bladder diaries. Finally, to confirm the clinical diagnosis of SUI, all participants were interviewed by an urotherapist via telephone. Any medical uncertainty was discussed, and if excluded, patients were contacted for medical advice and/or referral by one of the GPs in the project. Throughout the study, there was no face-to-face contact.

### Randomisation

Randomisation was through a pre-specified computer-generated list, in blocks of eight 22. An independent administrator kept the list and consecutively allocated eligible participants to one of the two intervention groups. There was no ‘blinding’ of group allocation to study participants, healthcare providers, or researchers.

### Intervention

Both groups had 3 months of treatment, via either an Internet-based programme or a programme sent by post. Both programmes included:

1. Information on SUI and associated lifestyle factors.
2. Pelvic floor muscle training.
3. Training reports (frequency, time spent).

Table 2 describes and contrasts the two interventions. More specifics for each intervention are given below.

**Table 2:** Description and comparison of the three months treatment programmes.

	Internet-based treatment programme	Postal treatment programme
Total extent, number of pages	20	8
Information, number of pages	9	4
Illustrations, *n*	33	7
Pelvic floor muscle training, design	Increasing intensity, login codes successively	Access to all exercises from start
Exercises (duration in s × repetitions × daily frequency):	Yes	Yes
– maximum contractions (for strength) (8 × 8–10 × 3)	Yes	Yes
– submaximal contractions (for endurance) (15–90 × 1 × 3)	Yes	Yes
– quick contractions (3 × 8–10 × 2–3)	Yes	Yes
– the ‘knack manoeuvre’*	Yes	Yes
Self-reported tests of progression	Yes	No
Training report	Once a week	At follow-up
Cognitive behavioural therapy assignments	Yes	No
E-mail support by urotherapist	Yes	No

* A conscious pelvic floor muscle contraction before and during physical stress.

### Internet-Based Treatment Programme

The programme contained eight escalating levels, and was modelled in line with other Internet-based interventions 23. Progress was self-monitored, with individually tailored support by a urotherapist. The intensity of the pelvic floor muscle training gradually increased. The urotherapist gave the participant login codes for two levels at a time, with instructions to maintain training at each level for at least 1 week. Every week, participants completed a self-evaluated test and reported a training diary to the urotherapist. New login codes were given with the passing of every other test, but not at a faster rate than every 2 weeks. In addition, the programme included cognitive behavioural therapy assignments for lifestyle change (if applicable), and for the identification and change in behaviours of avoidance and redundant security measures (if applicable).

Urotherapists actively contacted participants who failed to send in their reports according to schedule. Participants could contact their urotherapist at any time for support or questions. All contact was asynchronous, with encrypted e-mail, requiring a separate login from both participants and urotherapists. Response from the urotherapist was promised within 3 working days. Separate technical support was offered through encrypted e-mail contact with the website manager. The programme was built on a secure platform, using a two-factor authentication and Secure Sockets Layer (SSL), to provide communication security over the Internet. All parts of the programme could be downloaded for printing.

### Treatment Programme Sent by Post

In the print version, the first pages contained information, followed by instructions for pelvic floor muscle training. Participants were encouraged to increase the intensity of training successively, but had access to all exercises from the start. A training report was sent to the participants, for continuous registration throughout the treatment period, and it was returned together with the first follow-up. Participants in this group had no contact with the urotherapists.

### Outcome Measures

#### Primary outcomes

The mean symptom score was measured by the International Consultation on Incontinence Questionnaire Short Form (ICIQ-UI SF) 24. This instrument contains three items on frequency, amount of leakage, and overall impact on quality of life (QoL). Scoring is additive (0–21), with higher values indicating increased severity. The form also contains a fourth, non-scored item, used for the assessment of type of incontinence.

Condition-specific QoL was measured by the ICIQ-LUTSQoL 25–26. The instrument includes 19 items on the impact of leakage on role, physical, and social life, personal relationships, emotions, and sleep. All items are scored 1−4 (not at all/never, slightly/sometimes, moderately/often, a lot/all the time). Three items concerning personal relationships have an additional scoring alternative of ‘not applicable’. The overall score is 19−76, with higher values indicating increased impact on QoL.

#### Secondary outcomes

Patient global impression of improvement (PGI-I) 27 is a validated question asking the participants to rate their current condition compared to pre-treatment status. There are seven response options, ranging from ‘very much better’ to ‘very much worse’.

Health-specific QoL was evaluated with the EuroQol 5D-Visual Analogue Scale (EQ5D-VAS) 28, a vertical VAS with the endpoints 0 (worst imaginable health state) and 100 (best imaginable health state).

Incontinence episode frequency (IEF) was calculated from self-reported leakage episodes in the 2-day bladder diaries. A reduction in leakage episodes of >50% was considered clinically relevant 5.

Usage of UI aids was determined by asking participants to rate their usage of absorbent UI aids after treatment, compared with before treatment. Only those using UI aids before treatment were included in this analysis.

Satisfaction with the treatment programme was evaluated by asking participants to rate their experience of the programme. There were five response options, ranging from ‘very good’ to ‘very bad’.

### Sample Size

We based our power calculation on the primary outcome ICIQ-UI SF 29 and the secondary outcomes PGI-I 30 and IEF 15. The calculation for each outcome aimed to show a 20% difference between groups, with a power of 80% and a two-sided significance level of 0.05, allowing a dropout level of 20%. The resulting total sample sizes were 281 (ICIQ-UI SF), 203 (PGI-I), and 210 (IEF). For the ICIQ-UI SF, we anticipated a better effect in our study compared with the study protocol used for the calculations, because our participants would be younger and with pure SUI. Based on this, we decided to recruit a total of 250 participants (125 in each arm).

### Data Collection

Data was collected with postal self-assessed questionnaires and 2-day bladder diaries at baseline, and at follow-up performed 4 months after treatment initiation. We reminded non-respondents after 2 weeks by e-mail, after 4 weeks with a new questionnaire, and after 6 weeks by telephone. If no response was received after 8 weeks, participants were considered lost to follow-up.

### Statistical Analysis

To save overall scores in the ICIQ-UI SF and the ICIQ-LUTSQoL, we replaced missing answers at follow-up with the corresponding answer at baseline and vice versa in some questionnaires (ICIQ-UI SF, *n* = 6; ICIQ-LUTSQoL, *n* = 13). More than three missing answers in a row were considered deliberate, and left without action. When calculating the overall scores in the ICIQ-LUTSQoL, the answer ‘not applicable’ in questions concerning personal relationships was set to one, i.e. no impact. To obtain a weekly IEF measure, the values reported in the 2-day bladder diaries were multiplied by 3.5.

For baseline comparison of the two interventions groups, we used the Student’s *t*-test for continuous variables and the chi-square test for categorical variables. Treatment effects within groups were analysed using paired *t*-tests. For comparison of treatment effects between groups, we used a mixed model analysis for the primary outcomes and for health-specific QoL. However, this model could not be used for the IEF, where data was skewed with a high proportion of zeros. Instead, we analysed the IEF using a negative binomial regression. The remaining secondary outcomes, all single questions with ranked answers, were analysed using the Wilcoxon/Mann–Whitney rank sum test for differences between treatment groups. In addition, we calculated the effect sizes (mean standardised difference) with 95% CIs for each continuous measure. Effect sizes of >0.8 were considered large.

For additional analysis, the material was grouped by baseline UI severity, according to the overall score on the ICIQ-UI SF at inclusion (overall score 1–5, slight; 6–12, moderate; 13–18, severe; 19–21, very severe) 31.

A *P* < 0.05 was considered to indicate statistical significance. An intention-to-treat analysis was performed on all available data 32 using IBM-SPSS for Mac version 19.0 (IBM, Armonk, NY, USA).

### Ethics

The Regional Ethical Review Board, Umeå University approved the study (number 08-124M). Information about the study was given on our website. An informed consent form was included in the postal package sent for baseline investigation and was provided by all participants. No reimbursements were given. The study is registered at http://www.clinicaltrials.gov (ID: NCT01032265).

## Results

**Figure 1:**
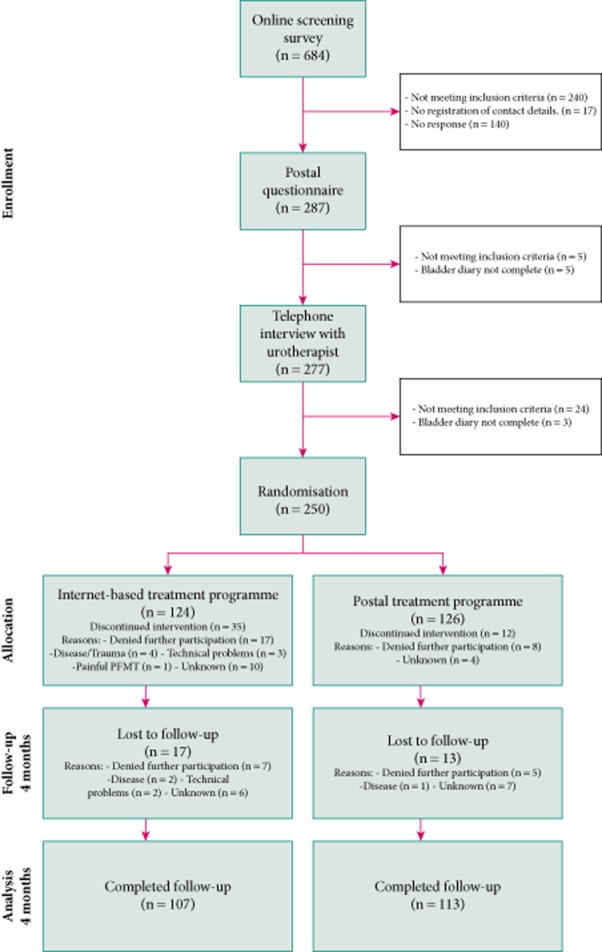
Flow of study participants.

The study was conducted in Sweden from December 2009 to April 2011. As expected, a large number of women completed the online screening survey, but several did not meet the inclusion criteria. Throughout the enrolment procedure, the most common reason for exclusion was UI other than SUI (40.1%, 174/434). Figure 1 shows the flow of study participants.

There were no significant differences between the treatment groups in baseline demographics, e.g. age, body mass index, education, nulliparity, menopausal status, or mean score on the ICIQ-UI SF and ICIQ-LUTSQoL at inclusion (Table 3).

**Table 3:** Baseline demographics and UI severity characteristics by treatment group

Variable	Internet-based treatment programme, *n* = 124	Postal treatment programme, *n* = 126	P*
Baseline demographics			
Mean (sd):			
Age, years	47.9 (10.6)	49.4 (9.8)	NS
BMI, kg/m^2^	24.7 (4.2)	24.5 (3.6)	NS
EQ5D-VAS score	79.1 (13.6)	79.2 (14.0)	NS
HADS score:			
Depression	2.2 (2.2)	2.3 (2.3)	NS
Anxiety	3.4 (2.6)	3.8 (3.2)	NS
* N* (%):			
Education:			
University level <3.0 years	25 (20.2)	28 (22.2)	NS
University level ≥3.0 years	63 (50.8)	72 (57.1)	NS
Daily smoker	4 (3.2)	5 (4.0)	NS
Nulliparous	9 (7.3)	7 (5.6)	NS
Postmenopausal	43 (35.8)	48 (39.7)	NS
Incontinence severity characteristics			
Mean (sd):			
ICIQ-UI SF score	10.4 (3.1)	10.3 (3.5)	NS
ICIQ-LUTSQoL score	33.6 (6.8)	33.6 (8.2)	NS

BMI, body mass index

* Based on Student’s *t*-test (means) or chi-square test (numbers).

Overall, 12.0% (30/250) of participants were lost to follow-up, 13.7% (17/124) from the Internet arm and 10.3% (13/126) from the postal arm. Compared with completers, participants lost to follow-up were significantly younger, had more severe leakage, and reported a larger impact on their condition-specific QoL at baseline (Table 4).

**Table 4:** Age and UI severity measures of participants lost to follow-up compared with completers

Variable	Lost to follow-up, *n* = 30	Completed follow-up, *n* = 220	P*
Baseline characteristics			
Mean (sd):			
Age, years	44.2 (9.2)	49.2 (10.2)	0.01
ICIQ-UI SF score	11.9 (3.9)	10.2 (3.2)	0.01
ICIQ-LUTSQoL score	37.2 (8.5)	33.1 (7.3)	0.01

* Student’s *t*-test.

### Primary Outcomes

Within both groups, there were highly significant improvements in the primary outcomes as assessed by ICIQ-UI SF and ICIQ-LUTSQoL. Table 5 reports overall scores, mean differences, and the effect size for each measure. The differences between groups were not significant.

**Table 5:** Summary of continuous outcome measures by treatment group. Values are the mean (sd) unless stated otherwise

Outcome variable	Treatment group	Baseline (*n* = 250)	4-month follow-up (*n* = 220)	Difference*	Within group *P*†	Between groups *P*‡	Effect size§ (95% CI)
Primary outcomes:							
ICIQ-UI SF	Internet	10.4 (3.1)	6.9 (3.1)	3.4 (3.4)	<0.001	0.27	0.99 (0.76–1.22)
		Postal	10.3 (3.5)	7.3 (3.9)	2.9 (3.1)	<0.001		0.95 (0.72–1.17)
ICIQ-LUTSQoL	Internet	33.6 (6.8)	27.8 (6.0)	4.8 (6.1)	<0.001	0.52	0.79 (0.57–1.01)
		Postal	33.6 (8.2)	28.8 (7.3)	4.6 (6.7)	<0.001		0.68 (0.47–0.89)
Secondary outcomes							
IEF	Internet	12.7 (12.0)	4.8 (7.7)	7.6 (9.1)	<0.001	0.23	0.84 (0.60–1.08)
		Postal	9.4 (8.6)	4.4 (6.7)	4.5 (7.1)	<0.001		0.63 (0.39–0.87)
EQ5D-VAS	Internet	79.1 (13.6)	83.3 (10.3)	3.7 (10.9)	0.001	0.30	0.34 (0.14–0.54)
		Postal	79.2 (14.0)	81.8 (13.9)	1.9 (13.0)	0.13		0.15 (–0.04 to 0.34)

* Based on participants with complete data on both occasions

† Based on paired t-tests

‡ Based on a mixed model analysis (ICIQ-UI SF, ICIQ-LUTS qol, and EQ5D-VAS), or a negative binomial regression (IEF)

§ Mean standardised difference.

**Figure 2:**
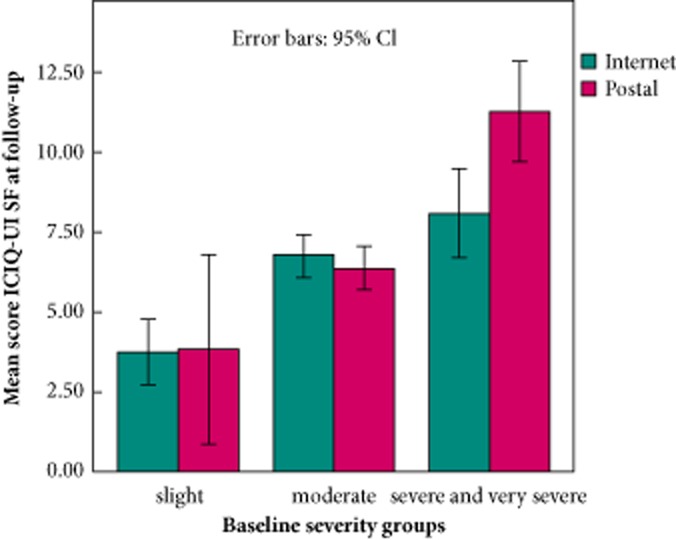
The mean ICIQ-UI SF scores at follow-up by baseline severity and treatment group.

Participants with severe leakage at baseline achieved a significantly lower mean score on the ICIQ-UI SF (mean score at follow-up 8.1 (95% CI 6.7–9.5) vs 11.0 (95% CI 9.4–12.5), *P* = 0.006) when treated with the Internet-based programme compared with the postal programme (Fig. 2).

### Secondary Outcomes

**Figure 3:**
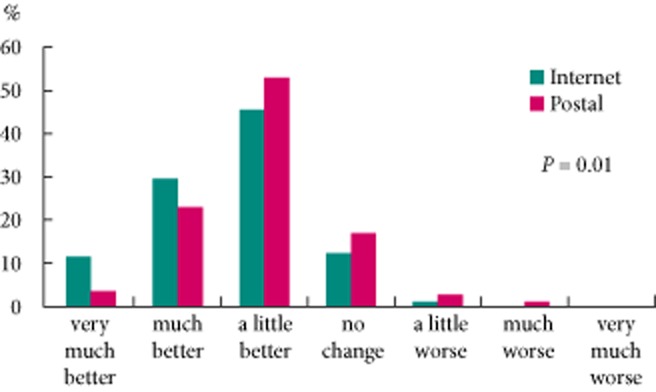
Distribution of responses on the PGI-I rating scale by treatment group. *P* value based on the Mann–Whitney rank sum test.

Analysis of the PGI-I showed that significantly more participants in the Internet group rated their leakage as much better or very much better after treatment (40.9%, 43/105, 95% CI 31.9–50.5), compared with participants in the postal group (26.5%, 30/113, 95% CI 19.0–35.3), *P* = 0.01 (Fig. 3).

Health-specific QoL (EQ5D) improved significantly in the Internet group (mean change 3.7 (95% CI 1.55–5.83), *P* = 0.001), but not in the postal group (mean change 1.9 (95% CI – 0.55 to 4.35), *P* = 0.13). However, the difference between groups was not significant (Table 5).

In both groups, the number of UI episodes per week (IEF) was significantly reduced. The mean reduction was significantly larger in the Internet group compared with the postal group (mean reduction 7.6 (95% CI 5.7–9.5) vs 4.5 (95% CI 2.9–6.0), *P* < 0.01), but when baseline values were taken into account, there was no significant difference between groups (Table 5). After treatment, 69.8% (120/172, 95% CI 62.6–76.3) of participants in both groups reported either complete absence of leakage or a reduction in leakage by >50% compared with baseline.

After treatment, more participants in the Internet group (59.5%, 47/79, 95% CI 48.4–69.9) than in the postal group (41.4%, 34/82, 95% CI 31.2–52.3), had either stopped using or reduced their usage of UI aids (*P* = 0.02).

In the Internet group, 84.8% (89/105, 95% CI 76.9–90.7) of participants experienced the treatment programme as ‘good’ or ‘very good’, compared with 62.9% (71/113, 95% CI 53.7–71.4) in the postal group (*P* < 0.001).

### Side-Effects

One woman in the Internet-group reported lower abdominal pain when conducting pelvic floor muscle training and discontinued her treatment. No other side-effects were reported.

## Discussion

In both the Internet-based and the postal treatment group, there were highly significant improvements with large effects sizes for symptom-score and condition-specific QoL. However, no significant differences were found between groups. Women with more severe leakage at baseline improved significantly more when treated with the Internet-based programme compared with the postal programme. The Internet-based treatment was also more effective for most secondary outcomes. Compared with the postal group, more women in the Internet group perceived their leakage as much or very much improved after treatment, more reported reduced usage of UI aids, and more indicated satisfaction with the treatment programme. Health-specific QoL improved in the Internet group but not in the postal group, and both groups had a clinically relevant reduction of leakage episodes.

### Strengths and Weaknesses of the Study

To our knowledge, this is the first randomised, controlled trial of Internet-based treatment for SUI. The clinical diagnosis is well substantiated and we compared two active treatments. Information provided to the participants was balanced and did not favour either of the treatments. During the study period there were no major technical problems or disruptions, and loss-to-follow up was low and similar between groups. Most outcome measures are established and recommended, and the research group included experienced GPs, urotherapists, and psychologists with broad knowledge on the topic. Limitations of the present study include that both treatment programmes were newly developed. The use of an established comparator would have been ideal, but there is currently no ‘gold standard’ for pelvic floor muscle training. A standardised face-to-face treatment or care-as-usual would have been an option, but we wanted the treatment programmes to be accessible for women from all over the country, even from remote areas or from areas with inadequate staffing. We also wanted to compare two simple and anonymous treatment alternatives, available to women that do not seek care because of lack of time, or because of embarrassment of their condition. In addition, the Internet-based treatment programme is a complex intervention and we cannot assess if any specific part of the programme is particularly important. Also, the programme required double log-ins from the participants, which was perceived as complicated by some women. A more simple technical solution might have lowered the discontinuation rate in the Internet group. Furthermore, it is possible that the study is underpowered. This is implied by all of the results favouring Internet treatment, although significant differences are not observed in some measures. We chose the outcome measures because we found them clinically relevant and well balanced for the evaluation of symptoms reported by women with SUI. However, at the time we made the power calculations there were few published studies using these measures, and the anticipated differences between the groups may have been overestimated. In addition, differences between the groups may have decreased as participants lost to follow-up had significantly more severe leakage, and those with severe leakage were unexpectedly seen to benefit more from the Internet-based treatment.

### Strengths and Weaknesses Compared with the Literature

Participants in the present study represent a clinically relevant group for a primary care setting, as they had moderate to severe leakage and all actively desired treatment. The wish for treatment is associated with the severity of the leakage and its impact on QoL 3–33, and is a prerequisite to succeed with a treatment completed on one’s own. Other influencing factors for improvement in the present study may be the capability to absorb written instructions, put them into practice, and for the Internet group to adequately use a computer. Although the treatment programmes were written in lay language and richly illustrated, the fact that our population was more highly educated than Swedish women in general may indeed have affected this capability. For comparison, 28% of the Swedish women aged 25−64 years had a university education of ≥3 years or in 2011 34. In the same year, a full 93% of the Swedish population had access to a computer with Internet connection, but frequent usage of the Internet is still higher among younger individuals and in higher socioeconomic class cohorts 18. Hence, the online recruitment might have limited our sample, and the results may not necessarily apply to a general population.

In both interventions, the minimum intensity of the training was the recommended eight contractions three times daily 7, but the pelvic floor muscle regimens were not exactly the same. The main difference was that the Internet group was supervised by urotherapists, whereas the postal group completed the training on their own. The interaction with the urotherapist may have influenced participants’ compliance and motivation to training, and improved the results in the Internet group. On the other hand, in the Internet programme the login codes for an escalating regimen were disclosed successively every second week, whereas in the postal programme participants had access to all types of exercises from the start. Consequently, participants in the postal group may have had a longer intense period of pelvic floor muscle training than participants in the Internet group.

The administration of a pamphlet for self-completion of pelvic floor muscle training is sometimes used as a sham treatment in clinical trials, and it could be argued that the improvements in the present study are merely placebo effects. However, the postal programme we used was extensive and the participants were informed that they received an active treatment. In addition, the improvements in the present study (mean change ICIQ-UI SF: Internet 3.4, postal 2.9) are of the same order of magnitude as in other studies on conservative management of SUI. For example, in a primary care setting in the Netherlands, where 384 participants with a baseline ICIQ-UI SF score of 11.2 were randomised to 3 months of either intense pelvic floor muscle training supervised by a nurse specialist or to care-as-usual, an improvement of the mean score by 2.0 was seen in the intervention arm 35. In an Australian study, 83 women with a mean age of 71.8 years and a baseline ICIQ-UI SF score of 10.4 improved their score by 3.0 after 3 months of pelvic floor muscle training, or by 1.3 after bladder training 36. In a study on duloxetine treatment, the active treatment arm obtained a 2.8 point improvement in the ICIQ-UI SF and the placebo arm improved by 1.7 points 37.

During a follow-up period of 4 months, some participants may have improved due to spontaneous remission. The annual remission rate of SUI has previously been calculated to be ≈7% 38. Based on this, about six women in our sample might have improved due to spontaneous remission, most likely with equal distribution in both groups.

### Meaning of the Study and Future Research

Despite the lack of significant differences between the groups in primary outcomes, there are many indications that the Internet treatment may be more effective than the postal programme. We also showed that it is possible to treat SUI without face-to-face contact. For the future, it is important to establish patient subgroups that benefit the most from each treatment, and how the programmes can best be integrated in everyday practice. Internet-based treatment may not be suitable for all women, but could facilitate access to care for some. It might also help unload primary healthcare, as costs are likely to be lower than for face-to-face treatments because the healthcare professionals can handle more patients in parallel. Even if efficacy is equal to or even lower than that of face-to-face treatments, the low delivery cost may make Internet-delivered treatment a more cost-effective alternative 39. The cost-effectiveness and the long-term effects of the treatments in the present study remain to be analysed, and will be reported in future articles.

### Conclusion

Management of SUI without face-to-face contact is possible, and may increase access to care. Internet-based treatment is a new, promising, and effective treatment alternative.

## Abbreviations

EQ5D-VAS: EuroQol 5D-Visual Analogue Scale
HADS: Hospital Anxiety and Depression Scale
ICIQ-UI SF: International Consultation on Incontinence Questionnaire Short Form
IEF: incontinence episode frequency
PGI-I: Patient global impression of improvement
QoL: quality of life
(S)UI: (stress) urinary incontinence
